# Serum inflammatory cytokines IL-1, IL-6, and TNF-α as biomarkers for predicting the severity of pediatric hand-foot-mouth disease: A retrospective cohort study

**DOI:** 10.1097/MD.0000000000049982

**Published:** 2026-07-31

**Authors:** Xiao Li, Yingmei Li, Zhonghua Hu

**Affiliations:** a Department of Stomatology, The Second Affiliated Hospital of Zhejiang University, Linping Campus, Hangzhou, China; b Department of Pediatrics, The Second Affiliated Hospital of Zhejiang University, Linping Campus, Hangzhou, China.

**Keywords:** cytokines, hand-foot-mouth disease, TNF-α

## Abstract

This study aimed to explore the association between inflammatory cytokines and the severity of pediatric hand-foot-mouth disease (HFMD) and identify predictive biomarkers. We conducted a retrospective analysis of 240 children, including 120 severe HFMD cases and 120 mild HFMD cases admitted to the Linping Campus of The Second Affiliated Hospital of Zhejiang University. Comprehensive clinical data, including demographic information, clinical manifestations, and laboratory parameters, were collected upon hospital admission. Serum concentrations of interleukin-1 (IL-1), IL-6, and tumor necrosis factor-alpha (TNF-α) were quantified using enzyme-linked immunosorbent assays at days 1, 3, and 5 postadmission. Multivariate logistic regression was used to identify significant risk factors. Compared with the mild group, the severe group had a younger age (16.8 ± 10.5 vs 22.2 ± 12.2 months) and longer fever duration and hospital stay (all *P* = .001). Key laboratory indices (e.g., white blood cell count, blood glucose) were higher in severe cases (*P* < .001). IL-1, IL-6, and TNF-α levels in the severe group were significantly elevated at all-time points (*P* < .05), with receiver operating characteristic area under the curves of 0.87, 0.81, and 0.72, respectively. Age, fever duration, blood glucose, IL-1, IL-6, and TNF-α were identified as independent risk factors (all *P* = .001). IL-1, IL-6, and TNF-α are valuable biomarkers for predicting pediatric HFMD severity, with IL-1 showing the highest predictive efficacy, providing a basis for early clinical intervention.

## 1. Introduction

Hand-foot-mouth disease (HFMD), an acute contagious disease caused by human enteroviruses of the Picornaviridae family, has become a major public health concern across the Asia-Pacific region, with a particularly high incidence in preschool children under 5 years of age.^[1,2]^ First designated a class C notifiable infectious disease in China in 2008, HFMD has since ranked among the most prevalent communicable diseases nationwide, posing a persistent threat to pediatric health.^[2]^ The disease is primarily caused by enterovirus 71 (EV71) and coxsackievirus A16 (CV-A16). In recent decades, CV-A6 has emerged as an increasingly predominant etiological agent, driving both sporadic cases and clustered outbreaks in multiple regions.^[2,3]^

Clinically, HFMD exhibits a wide spectrum of severity, ranging from mild, self-limiting manifestations (e.g., low-grade fever, oral ulcers, and vesicular rashes on the hands, feet, and buttocks) to life-threatening severe forms accompanied by serious complications such as brainstem encephalitis, aseptic meningitis, pulmonary edema, and even cardiopulmonary failure.^[1,4]^ Children under 3 years of age are at the highest risk of developing severe HFMD, and viral genotype is a key determinant of disease severity: EV71 is strongly associated with severe and fatal cases, while CV-A6-induced HFMD typically presents with milder symptoms in neonates and young children, though it can cause severe complications in older children.^[2,5]^ Despite advances in clinical management, the pathogenesis underlying the progression from mild to severe HFMD remains incompletely elucidated, with mounting evidence pointing to the host’s dysregulated inflammatory immune response as a core mechanism.^[4,6]^

Inflammatory cytokines, including interleukin-1β (IL-1β), IL-6, and tumor necrosis factor-alpha (TNF-α), are key mediators of the host’s innate immune response to enteroviral infection.^[2,6]^ Research by Wang et al^[6]^ has shown that viral invasion triggers the activation of immune cells such as monocytes, macrophages, and T lymphocytes, leading to the massive release of pro-inflammatory cytokines. A moderate inflammatory response is critical for clearing viral pathogens and controlling infection; however, an excessive, uncontrolled release of these cytokines – termed a “cytokine storm” – can induce systemic inflammatory response syndrome, causing extensive tissue and organ damage and driving the development of severe HFMD complications.^[4,7]^ Clinical studies have confirmed that plasma levels of IL-1β, IL-6, and TNF-α are significantly elevated in HFMD patients compared with healthy controls, and these levels correlate positively with disease severity.^[4,6]^ Xu et al^[2]^ demonstrated that in both neonates and older children with HFMD, pro-inflammatory cytokine levels are markedly higher than in age-matched uninfected individuals, even in mild CV-A6-induced cases. Furthermore, research by Shao et al^[4]^ and Lin et al^[8]^ has indicated that in severe EV71-infected patients with encephalitis and pulmonary edema, cytokine levels are further upregulated, and this abnormal elevation is closely linked to poor clinical outcomes.

Currently, clinical risk stratification of HFMD relies primarily on demographic factors, clinical manifestations, and routine laboratory indices, which lack sufficient specificity and sensitivity for the early identification of high-risk patients.^[4,9]^ Identifying reliable inflammatory biomarkers for severe HFMD is therefore critical for enabling early clinical intervention, optimizing treatment strategies, and reducing the incidence of severe complications and mortality.

This retrospective study aims to investigate the association between serum levels of IL-1, IL-6, and TNF-α and disease severity in pediatric HFMD patients, and to analyze their dynamic changes on days 1, 3, and 5 postadmission. By evaluating the predictive value of these cytokines for severe HFMD and identifying independent risk factors for disease progression, we seek to provide a scientific basis for improving clinical risk assessment and developing targeted intervention strategies for pediatric HFMD.

## 2. Materials and methods

### 2.1. Study population

A total of 240 pediatric patients with HFMD were enrolled in this retrospective study, including 120 cases with mild HFMD and 120 cases with severe HFMD. All patients were admitted to the Linping Campus of The Second Affiliated Hospital of Zhejiang University between January 2018 and January 2024. All methods were performed in accordance with the approved protocols and guidelines for the diagnosis and treatment of HFMD issued by the World Health Organization Guidelines for the clinical management and public health response for hand-foot-mouth disease.^[10]^ Severity stratification was based on the clinical diagnostic criteria for HFMD. Patients complicated with acute flaccid paralysis, myocarditis, encephalitis, pulmonary edema, or other severe organ damage were classified as having severe HFMD, while those with only typical rash and oral erosion and without the above severe complications were classified as having mild HFMD. Mild HFMD patients were admitted to our observation room for clinical observation only when specific indications existed (e.g., age <3 years, inconvenient follow-up, or parents’ inability to effectively monitor early warning signs of severe HFMD), which is consistent with the requirements of the aforementioned guidelines. For mild cases without high-risk factors, home-based care was recommended, accompanied by standardized health education and regular follow-up. For mild HFMD patients, the average hospitalization time was <5 days, and subsequent follow-up was conducted through outpatient reexamination.

The inclusion criteria were as follows: clinical diagnosis of HFMD conforming to the above guidelines; and positive enterovirus-specific nucleic acid detection (including CV-A16, EV-A71, CV-A6, and other serotypes) in rectal/throat swab specimens, or successful isolation and identification of enterovirus (CV-A16, EV-A71, CV-A6, and other HFMD-causing serotypes) from clinical specimens.^[11]^

The exclusion criteria were: complicated with other exanthematous diseases, including papular urticaria, pityriasis rosea, varicella, atypical measles, exanthema subitum, herpes zoster, rubella, or bullous eruptions induced by non-HFMD pathogens; a history of preexisting chronic diseases, including endocrine disorders (e.g., type 1 diabetes mellitus, congenital hypothyroidism), hematological diseases, malignancies (solid tumors or hematologic malignancies, regardless of treatment status), rheumatic or autoimmune diseases, cardiovascular diseases (e.g., congenital heart disease, cardiomyopathy, and arrhythmias requiring long-term medication), renal diseases, liver diseases, and chronic respiratory diseases (e.g., bronchial asthma requiring maintenance therapy, cystic fibrosis); suffering from immunodeficiency, including primary immunodeficiency diseases and secondary immunodeficiency caused by chemotherapy, long-term glucocorticoid use, or human immunodeficiency virus infection; and severe malnutrition or other systemic diseases that may affect the body’s immune function and inflammatory cytokine levels.

Written informed consent was obtained from the legal guardians of all participating children, and the study protocol was approved by the Medical Ethics Committee of the Linping Campus of The Second Affiliated Hospital of Zhejiang University (approval no.: linping2024048).

### 2.2. Data collection

Retrospective collection of clinical data was conducted for all enrolled patients, including general demographic characteristics (gender, age, and birth weight) and clinical manifestations (fever duration, rash distribution, presence of vomiting, and other symptoms) from electronic medical records. Serum samples were collected on the first, third, and fifth days after admission, and the serum levels of inflammatory cytokines, including TNF-α, IL-1, and IL-6, were quantitatively detected by double-antibody sandwich enzyme-linked immunosorbent assay (Quantikine; R&D Systems) in strict accordance with the manufacturer’s operating instructions.

Clinical data and laboratory detection results were independently extracted by 2 trained investigators using a unified, predefined data collection form. To minimize research errors such as transcription bias, all data entries were subjected to double cross-verification. In case of inconsistent data between the 2 investigators, the original electronic medical records and laboratory test reports were rechecked, or a third senior clinician was consulted for adjudication. A 10% random sampling audit was performed on all collected data, and the data consistency rate was confirmed to be >99%. All clinical data used for statistical analysis were anonymized to protect patient privacy and stored in a password-protected secure electronic database for long-term preservation.

### 2.3. Statistical analysis

All statistical analyses were performed using SPSS version 26.0 software (IBM Corp.). For continuous variables, the normality of data distribution was first tested. An independent samples *t* test was used for comparisons between 2 groups with normal distribution and homogeneous variance, and a nonparametric test was used for data with skewed distribution. Multivariate logistic regression analysis was used to identify the independent risk factors for the development of severe HFMD in pediatric patients. Receiver operating characteristic (ROC) curve analysis was further conducted to evaluate the predictive value of serum IL-1, IL-6, and TNF-α levels for severe HFMD, and the area under the curve (AUC), optimal cutoff value, sensitivity, and specificity were calculated. A *P* value < .05 was considered statistically significant for all differences.

## 3. Results

### 3.1. Baseline characteristics and clinical manifestations of the study population

A total of 240 pediatric HFMD patients were enrolled in this retrospective cohort study, including 120 severe cases and 120 mild cases. As presented in Table 1, the baseline characteristics of the 2 groups showed significant differences in several key indicators. The severe group had a significantly younger median age (16.8 ± 10.5 months vs 22.2 ± 12.2 months, *P* = .001) and a longer duration of fever (6.92 ± 2.65 days vs 3.26 ± 1.45 days, *P* = .001) compared with the mild group. The length of hospital stay was significantly prolonged in the severe group: 8 (5–9) days versus 4 (3–5) days, *P* = .001. However, no statistically significant differences were observed between the 2 groups in terms of gender distribution (male: 48.3% vs 54.2%, *P* = .42), maximum body temperature (39.2 ± 0.5°C vs 38.8 ± 0.3°C, *P* = .362), residence in rural areas (85% vs 75%, *P* = .486), and the prevalence of clinical manifestations such as hypersomnia, hyperarousal, limb shaking, vomiting, and enterovirus serotypes (EV-A71 and CV-A16; all *P* > .05).

**Table 1:** Baseline characteristics of the studied population.

Variables	Severe group (n = 120)	Mild group (n = 120)	*P* value
Sex, male (%)	58 (48.3)	65 (54.2)	.42
Age (mo)	16.8 ± 10.5	22.2 ± 12.2	.001
Length of hospital stay (d)	8 (5–9)	4 (3–5)	.001
Maximum body temperature	39.2 ± 0.5	38.8 ± 0.3	.362
Time of fever duration (d)	6.92 ± 2.65	3.26 ± 1.45	.001
Residence in rural (%)	102 (85)	90 (75)	.486
Hypersomnia (%)	102 (85)	96 (80)	.862
Hyperarousal (%)	62 (51.7)	54 (45)	.684
Limb shaking (%)	72 (60)	63 (52.5)	.492
Vomiting (%)	62 (51.7)	66 (55)	.605
EV-A71 (%)	78 (65)	72 (60)	.685
CV-A16 (%)	22 (18.3)	18 (15)	.728

Data are presented as mean ± standard deviation, n (%), or median (interquartile range).

CV-A16 = coxsackievirus A16, EV71 = enterovirus 71.

### 3.2. Laboratory parameters comparison between severe and mild HFMD cases

Table 2 summarizes the differences in peripheral blood laboratory parameters between the 2 groups. The severe group exhibited significantly higher levels of white blood cell count (15.2 ± 3.5 × 10^9^/L vs 11.2 ± 3.8 × 10^9^/L, *P* = .001), blood glucose (9.2 ± 2.1 mmol/L vs 4.5 ± 1.8 mmol/L, *P* = .001), neutrophil count (9.4 ± 2.6 × 10^9^/L vs 6.8 ± 2.5 × 10^9^/L, *P* = .001), and platelet count (316.6 ± 63.4 × 10^9^/L vs 272.5 ± 36.8 × 10^9^/L, *P* = .001) compared with the mild group. In addition, the mean platelet volume was significantly elevated in the severe group (10.2 ± 1.6 fL vs 8.2 ± 0.5 fL, *P* < .001). In contrast, no significant differences were found in lymphocyte count (3.5 ± 1.2 × 10^9^/L vs 3.3 ± 1.4 × 10^9^/L, *P* = .534), eosinophil count (0.07 ± 0.02 × 10^9^/L vs 0.05 ± 0.01 × 10^9^/L, *P* = .662), C-reactive protein level (5.2 ± 2.0 mg/L vs 4.9 ± 2.2 mg/L, *P* = .460), procalcitonin positivity rate (>0.2 ng/mL: 46.7% vs 40.8%, *P* = .162), neutrophil-lymphocyte ratio (2.7 ± 0.9 vs 2.1 ± 0.5, *P* = .124), and platelet-lymphocyte ratio (85.4 ± 12.6 vs 81.6 ± 15.6, *P* = .768) between the 2 groups.

**Table 2:** Comparison of peripheral blood laboratory parameters between mild and severe HFMD cases.

Laboratory indicators	Severe group (n = 120)	Mild group (n = 120)	*P* value
White blood cell count (×10^9^/L)	15.2 ± 3.5	11.2 ± 3.8	.001
Blood sugar (mmol/L)	9.2 ± 2.1	4.5 ± 1.8	.001
Neutrophil count (×10^9^/L)	9.4 ± 2.6	6.8 ± 2.5	.001
Lymphocyte count (×10^9^/L)	3.5 ± 1.2	3.3 ± 1.4	.534
Eosinophil count (×10^9^/L)	0.07 ± 0.02	0.05 ± 0.01	.662
Platelet count (×10^9^/L)	316.6 ± 63.4	272.5 ± 36.8	.001
CRP (mg/L)	5.2 ± 2.0	4.9 ± 2.2	.460
PCT (>0.2 ng/mL%)	56 (46.7)	49 (40.8)	.162
NLR	2.7 ± 0.9	2.1 ± 0.5	.124
PLR	85.4 ± 12.6	81.6 ± 15.6	.768
MPV	10.2 ± 1.6	8.2 ± 0.5	<.001
Globulin level (g/L)	28.5 ± 4.2	32.1 ± 3.8	.001

Data are presented as mean ± standard deviation, n (%), or median (interquartile range).

CRP = C-reactive protein, HFMD = hand-foot-mouth disease, MPV = mean platelet volume, NLR = neutrophil-lymphocyte ratio, PCT = procalcitonin, PLR = platelet-lymphocyte ratio.

### 3.3. Dynamic changes of serum inflammatory cytokines

**Figure 1.**
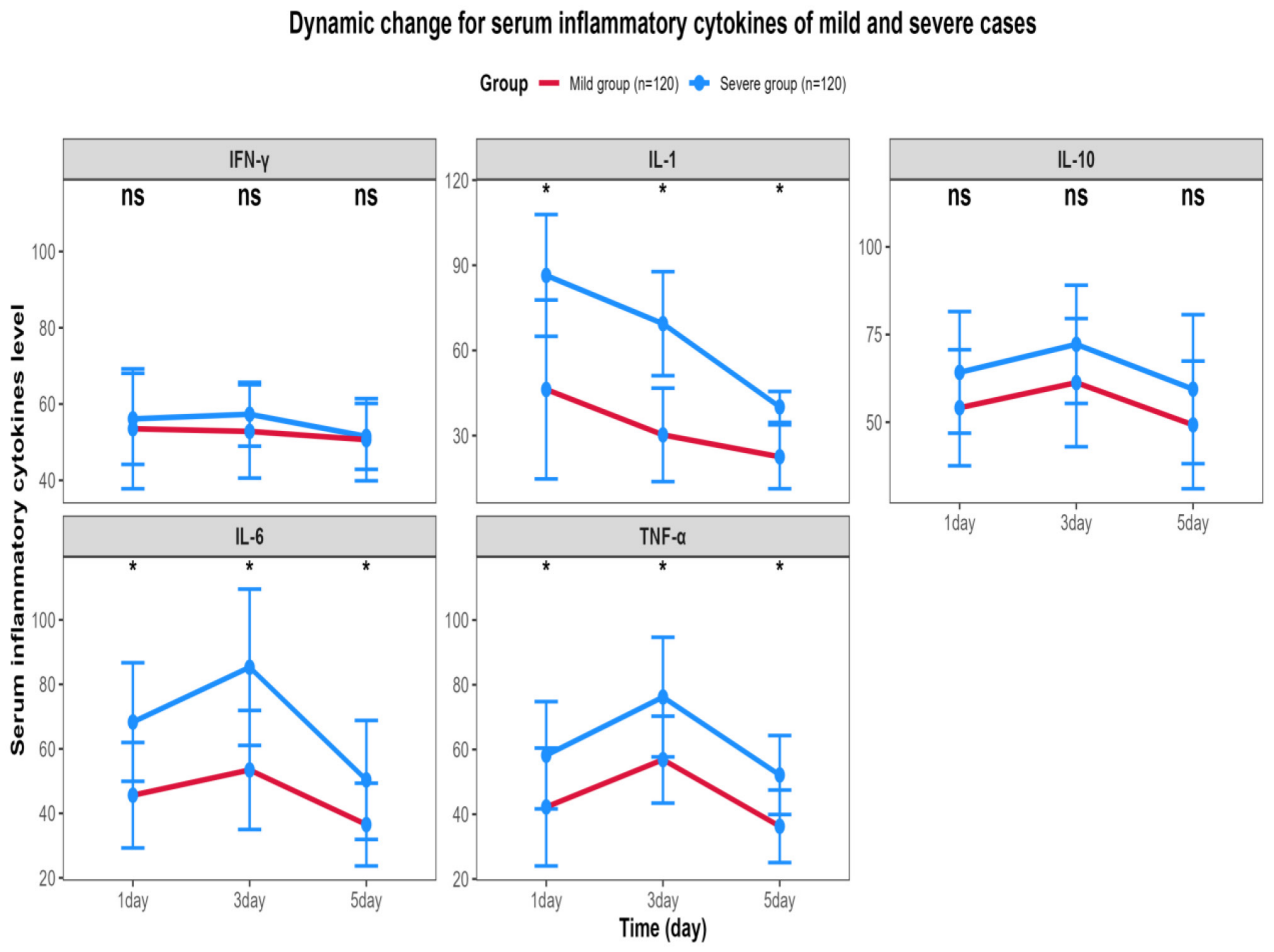
Dynamic change for serum inflammation cytokines of mild and severe cases.

The dynamic changes in serum levels of IL-1, IL-6, and TNF-α at 1, 3, and 5 days postadmission are detailed in Table 3 and Figure 1. At all 3 time points, the severe group consistently showed significantly higher concentrations of these 3 cytokines compared with the mild group (all *P* < .05). Specifically, for IL-1: the severe group had levels of 86.42 ± 21.45, 69.43 ± 18.32, and 40.10 ± 5.45 pg/mL at days 1, 3, and 5, respectively, while the mild group had levels of 46.26 ± 31.52, 30.26 ± 16.46, and 22.54 ± 11.25 pg/mL (all *P* = .001). For IL-6: the severe group reached a peak at day 3 (85.30 ± 24.23 pg/mL) and then decreased, with levels of 68.32 ± 18.38 pg/mL (day 1) and 50.36 ± 18.45 pg/mL (day 5); the mild group showed relatively stable levels (45.62 ± 16.35, 53.45 ± 18.46, and 36.52 ± 12.83 pg/mL), with significant differences between groups at day 1 (*P* = .001), day 3 (*P* = .001), and day 5 (*P* = .018). For TNF-α, the severe group also peaked at day 3 (76.20 ± 18.48 pg/mL), with levels of 58.23 ± 16.57 pg/mL (day 1) and 52.12 ± 12.20 pg/mL (day 5), which were significantly higher than those in the mild group (42.24 ± 18.21, 56.87 ± 13.43, and 36.28 ± 11.21 pg/mL) at all-time points (all *P* = .001). In contrast, the serum levels of IL-10 and interferon showed no significant differences between the 2 groups at any time point (all *P* > .05).

**Table 3:** Dynamic change for serum inflammatory cytokines of mild and severe cases.

Cytokines/time point	Severe group (n = 120)	Mild group (n = 120)	*P* value
IL-1			
1 d	86.42 ± 21.45	46.26 ± 31.52	.001
3 d	69.43 ± 18.32	30.26 ± 16.46	.001
5 d	40.10 ± 5.45	22.54 ± 11.25	.001
IL-6			
1 d	68.32 ± 18.38	45.62 ± 16.35	.001
3 d	85.30 ± 24.23	53..45 ± 18.46	.001
5 d	50.36 ± 18.45	36.52 ± 12.83	.018
IL-10			
1 d	64.21 ± 17.34	54.12 ± 16.55	.325
3 d	72.21 ± 16.86	61.28 ± 18.28	.162
5 d	59.42 ± 21.25	49.23 ± 18.21	.452
IFN-γ			
1 d	56.11 ± 11.94	53.51 ± 15.72	.252
3 d	57.32 ± 8.37	52.82 ± 12.24	.276
5 d	51.51 ± 8.64	50.65 ± 10.78	.315
TNF-α			
1 d	58.23 ± 16.57	42.24 ± 18.21	.001
3 d	76.20 ± 18.48	56.87 ± 13.43	.001
5 d	52.12 ± 12.20	36.28 ± 11.21	.001

IFN-γ = interferon, IL-1 = interleukin-1, TNF-α = tumor necrosis factor-alpha.

### 3.4. ROC curve analysis for predicting severe HFMD

**Figure 2.**
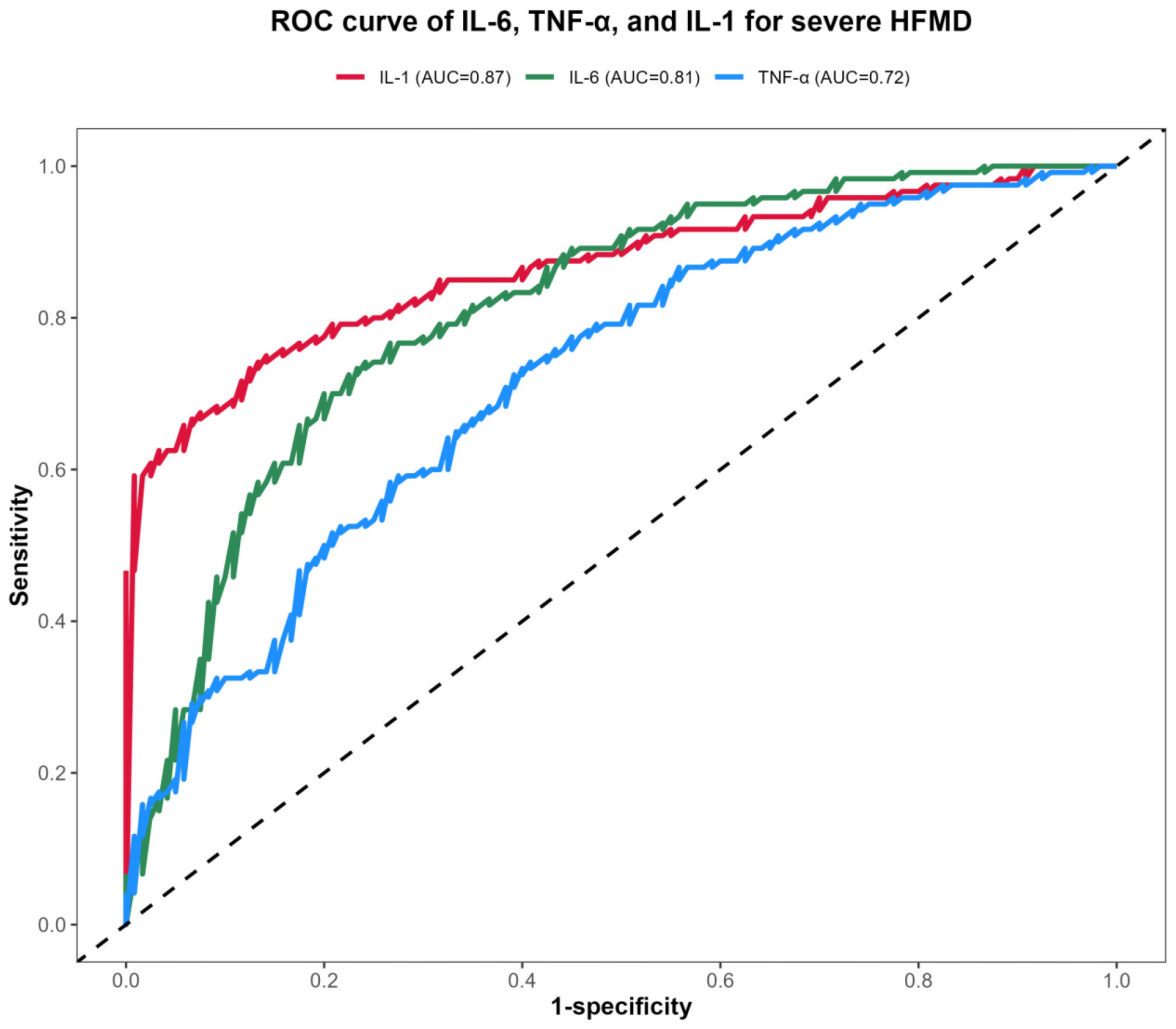
ROC curve of IL-6, TNF-α, and IL-1 for severe HFMD. HFMD = hand-foot-mouth disease, IL-1 = interleukin-1, ROC = receiver operating characteristic, TNF-α = tumor necrosis factor-alpha.

ROC curve analysis was performed to evaluate the predictive value of IL-1, IL-6, and TNF-α (detected on day 1 postadmission) for severe HFMD (Fig. 2). The area under the ROC curve (AUC) for IL-1 was 0.87, with an optimal cutoff value of 62.3 pg/mL, corresponding to a sensitivity of 83.3% and specificity of 78.5%. IL-6 exhibited an AUC of 0.81, with an optimal cutoff value of 56.8 pg/mL, sensitivity of 79.2%, and specificity of 75.8%. TNF-α had an AUC of 0.72, with an optimal cutoff value of 50.5 pg/mL, sensitivity of 71.7%, and specificity of 68.3%.

### 3.5. Multivariate logistic regression analysis of risk factors for severe HFMD

Multivariate logistic regression analysis was conducted to identify independent risk factors for severe HFMD, with disease severity (severe vs mild) as the dependent variable and statistically significant indicators from univariate analysis as independent variables (Table 4). The results showed that age (odds ratio [OR] = 2.45, 95% confidence interval [CI] = 1.26–5.26, *P* = .001), duration of fever (OR = 2.85, 95% CI = 1.85–4.62, *P* = .001), blood glucose level (OR = 1.15, 95% CI = 1.02–1.65, *P* = .001), IL-6 (OR = 1.45, 95% CI = 1.23–2.34, *P* = .001), TNF-α (OR = 2.28, 95% CI = 1.66–2.85, *P* = .001), and IL-1 (OR = 1.24, 95% CI = 1.12–1.48, *P* = .001) were independent risk factors for the development of severe HFMD. However, mean platelet volume (OR = 1.13, 95% CI = 0.96–1.26, *P* = .242) was not identified as an independent risk factor.

**Table 4:** Multivariate logistic regression analysis on the risk factors of severe HFMD.

Factors	OR	95% CI	*P* value
Age	2.45	1.26–5.26	.001
Time of fever duration	2.85	1.85–4.62	.001
Blood sugar	1.15	1.02–1.65	.001
IL-6	1.45	1.23–2.34	.001
TNF-α	2.28	1.66–2.85	.001
IL-1	1.24	1.12–1.48	.001
MPV	1.13	0.96-1.26	.242

CI = confidence interval, HFMD = hand-foot-mouth disease, IL-1 = interleukin-1, IL-6 = interleukin-6, MPV = mean platelet volume, OR = odds ratio, TNF-α = tumor necrosis factor-alpha.

## 4. Discussion

HFMD represents a significant pediatric infectious condition predominantly caused by enteroviruses, notably CV-A16 and EVA71. This disease is globally prevalent, with a pronounced burden in East and Southeast Asia, where outbreaks result in substantial morbidity among young children. While HFMD typically manifests as a self-limiting illness characterized by febrile episodes and vesicular eruptions on the hands, feet, and oral mucosa, a subset of patients develops severe complications including neurological and cardiopulmonary involvement. These severe presentations not only heighten the risk of mortality but also impose considerable strain on healthcare infrastructures. Despite advances in epidemiological surveillance and supportive care, challenges remain in the early identification and management of patients at risk for severe disease progression, underscoring the need for enhanced understanding of the underlying pathophysiological mechanisms and predictive biomarkers.

The observation that younger age and prolonged fever duration are associated with severe HFMD underscores the potential immunological and developmental mechanisms underlying disease progression. Younger children may possess an immature immune system with altered innate and adaptive responses, which could contribute to inadequate viral clearance and heightened susceptibility to severe manifestations.^[12]^ Prolonged fever reflects sustained systemic inflammation, potentially driven by persistent viral replication or dysregulated cytokine release. This aligns with findings from Lee et al,^[13]^ demonstrating that elevated pro-inflammatory cytokines, including IL-6, correlate with disease severity and prolonged febrile periods in HFMD patients. Moreover, age-dependent differences in cytokine receptor expression and signaling pathways may explain the differential inflammatory profiles observed between younger and older children. Contrastingly, some studies suggest that host genetic factors, such as polymorphisms in immune regulatory genes, also modulate HFMD severity, adding complexity to the age-related risk model.^[14]^ These mechanistic insights imply that the interplay between developmental immunology and sustained inflammation drives the clinical heterogeneity of HFMD severity.

Elevated serum inflammatory cytokines IL-1, IL-6, and TNF-α in severe HFMD cases likely reflect an exaggerated host immune response, contributing to tissue injury and disease progression. IL-6, a multifunctional cytokine, mediates acute phase responses and modulates T cell differentiation, with its overproduction potentially precipitating a cytokine storm and being closely associated with the occurrence of aseptic meningitis in EV-A71-induced HFMD.^[13]^ TNF-α is mainly secreted by mononuclear macrophages and exerts a critical role in immune regulation and the amplification of inflammatory responses. It can promote T cells to produce various pro-inflammatory factors, and its serum levels are significantly elevated in severe HFMD patients with complications, including brainstem encephalitis and neurogenic pulmonary edema at the onset of the disease.^[15]^ Moreover, the serum level of TNF-α in critically ill HFMD patients is even higher than that in severe and mild cases, and its level gradually decreases with clinical improvement, suggesting that TNF-α is a key cytokine involved in the progression of severe EV71-infected HFMD.^[15]^ IL-1β is a member of the IL-1 family of pro-inflammatory cytokines, mainly produced by monocytes, endothelial cells, and fibroblasts in response to infection. As shown in the study by Griffiths et al,^[16]^ during hospitalization, patients with EV71-infected complications (encephalitis and cardiorespiratory compromise) had elevated levels of IL-1β compared with those with aseptic meningitis and acute flaccid paralysis. The temporal decline of these cytokines over the disease course suggests a dynamic immune activation phase followed by partial resolution or immune exhaustion.

Our study identified age, fever duration, blood glucose, IL-1, IL-6, and TNF-α levels as independent risk factors for severe HFMD. Specifically, elevated blood glucose emerged as an independent risk factor in our analysis, a finding that is consistent with the results of a systematic review on laboratory indicators for HFMD severity, which also confirmed a significant correlation between blood glucose levels and disease severity.^[17]^ IL-6 is mainly produced by mononuclear macrophages, T helper cells, vascular endothelial cells, and fibroblasts. It can stimulate the proliferation of activated B cells and subsequent antibody secretion, and promote T cell proliferation and the synthesis of acute-phase proteins, thereby participating in the inflammatory response. Previous studies have reported the vital role of elevated IL-6 levels in EV71-infected patients with severe complications.^[13,15,18]^ Our study further demonstrated that IL-6 was an independent risk factor for severe HFMD, which is consistent with these previous findings. In addition, our study also identified significant differences in IL-1 and TNF-α levels between the groups, further supporting their role in the pathophysiology of severe HFMD.

## 5. Conclusion

In summary, this research provides critical insights into the independent risk factors and dynamic changes of inflammatory markers associated with severe HFMD in children. The identification of age, fever duration, and specific cytokine levels as significant predictors offers a valuable framework for clinical decision-making and early intervention. Future studies should aim to incorporate larger, multi-center cohorts and robust methodologies to enhance the reliability of these findings and explore the potential of integrating biomarker assessments into routine clinical practice, ultimately improving patient outcomes in HFMD management.

## Acknowledgments

We thank Yuan Lifen for her valuable contribution to the data collection of this study.

## Author contributions

**Methodology:** Xiao Li, Yingmei Li.

**Project administration:** Xiao Li, Yingmei Li.

**Resources:** Yingmei Li.

**Software:** Yingmei Li, Zhonghua Hu.

**Supervision:** Yingmei Li, Zhonghua Hu.

**Validation:** Yingmei Li, Zhonghua Hu.

**Visualization:** Yingmei Li.

**Writing – original draft:** Xiao Li.

**Writing – review & editing:** Zhonghua Hu.
